# Antimicrobial properties of promising Zn–Fe based layered double hydroxides for the disinfection of real dairy wastewater effluents

**DOI:** 10.1038/s41598-023-34488-y

**Published:** 2023-05-10

**Authors:** Sahar Abdel Aleem Abdel Aziz, Yasser GadelHak, Manar Bahaa El Din Mohamed, Rehab Mahmoud

**Affiliations:** 1 Department of Hygiene, Zoonoses and Epidemiology, Faculty of Veterinary Medicine, Beni-Suef University, Beni Suef, 62511 Egypt; 2 Department of Materials Science and Nanotechnology, Faculty of Postgraduate Studies for Advanced Sciences, Beni-Suef University, Beni Suef, 62511 Egypt; 3 Department of Chemistry, Faculty of Science, Beni-Suef University, Beni Suef, 62511 Egypt

**Keywords:** Microbiology techniques, Nanoscale materials

## Abstract

Bacterial resistance to conventional antibiotics is a serious challenge that requires novel antibacterial agents. Moreover, wastewater from dairy farms might contain countless number of pathogens, organic contaminants and heavy metals that consider a threat to the terrestrial and aquatic environment. Therefore, the development of cost-effective, highly operation-convenient, recyclable multifunctional antimicrobial agents became an urgent necessity. Layered double hydroxides (LDH) have shown promising results as antibacterial agents. However, more work is required to further investigate and improve the antimicrobial performance of LDH structures against pathogens. In this study three Zn–Fe based LDH were investigated for real dairy wastewater disinfection. The three LDH samples were cobalt substituted Zn–Fe LDH (CoZnFe), magnesium substituted Zn–Fe LDH (MgZnFe) and MgZnFe-Triazol LDH (MgZnFe-Tz) nanocomposite. Seventy-five wastewater samples were collected from a dairy farm sewage system. The sensitivity of isolated pathogens was tested against two commonly used disinfectants (Terminator and TH4) and was assessed against the three LDH samples at different concentrations. The overall prevalence of S. *agalactiae*, S. *dysgalactiae* and *Staph. aureus* was significantly at 80.0% (P-value = 0.008, X2 = 9.700). There was variable degree of resistance to the tested disinfectants, whereas the antimicrobial activity of CoZnFe LDH was increased significantly at a concentration of 0.005 mg/L followed by MgZnFe LDH while MgZnFe-Tz LDH showed minor antibacterial potency. It was concluded that CoZnFe LDH showed a better biocidal activity in killing the isolated resistant pathogens, making it a good choice tool in combating the zoonotic microbes in wastewater sources.

## Introduction

Over the last decades, and due to the scarcity of water resources, the need for improved water reclamation technologies^1^ has risen so that wastewater is treated to meet standard water reuse quality criteria^2^. Wastewater may contain countless number of bacteria including coliforms, *Streptococci (S.)*, *Staphylococci* (*Staph*.), *Proteus* group, anaerobic sporefroming bacteria and many other types of pathogens^3^. Wastewater has proved to contain many zoonotic bacteria that possess a great public health risk to human communities^4^. The process of wastewater treatment depends on several points such as wastewater composition, biological oxygen demand, pH, presence of toxic compounds and others^5^.

One of the main sources of wastewater effluents is the dairy industry. To meet the ever rising demand from customers, milk output has significantly grown. However, the rising dairy sector added more environmental stressors in the form of waste products produced. The resulting toxic materials, which are often emitted as solids, liquid effluent, and slurries and contain a variety of organic and inorganic compounds, are dangerous to human health and have an adverse effect on the development of flora and fauna. Dairy effluents contain a high organic load, which is to blame for the receiving streams' rapidly declining dissolved oxygen (DO) levels. They also serve as a breeding site for mosquitoes and flies that spread diseases including dengue fever, malaria and yellow fever, in addition to several food-borne bacteria through the mechanical transmission^6^. Numerous dairy farms can produce water contaminants that are harmful to aquatic life, including fat, milk proteins, lactose, lactic acid, minerals, and detergents and sanitizers^7^. Dairy effluents contain nutrients like nitrogen that cause the receiving water bodies causing eutrophication, while detergents and sanitizers have an impact on aquatic life^8^. Additionally, because it can be converted to nitrate, nitrogen from dairy wastes can also affect ground water^9^. Ammonia, nitrite, and nitrate are all types of nitrogen that are bad for human health^10^. Additionally, nitrite is also notorious to cause intestinal cancer and nitrate is responsible for methanoglobinemia if converted to nitrite^11^.

Pretreatment of dairy wastewater is critical step before biological treatment to eliminate various contaminants. Dairy wastewater disinfection is one of the steps required for the pretreatment of such effluents. The most common contagious pathogens *Staph. aureus*, *S. agalactiae*, and *S. dysgalactiae*, which have adapted to survive inside the mammary gland and are, spread from cow to cow at or around the time of milking, cause both acute clinical and persistent subclinical mastitis. This causes significant financial loss to the dairy industry including impacts on the milk quantity and quality, culling of infected cows and treatment costs^12,13^. Moreover, the subclinical mastitis is frequently asymptomatic the most prevalent type, once established, many of these illnesses last the duration of the animal's life or even entire the lactation period, absence of methods for their recognition also, it is considered a reservoir of microorganisms that can be transmitted to other animals within the farm or even others^14^.

Due to the complex nature of wastewater strains, bacteria disenfection requires the development of advanced multifunctional nano-adsorbents that have excellent antimicrobial properties^15^. Numerous types of nanomaterials were investigated for their antibacterial effects against gram-positive and gram-negative bacteria^16–18^. One of the promising types of nanomaterials that are less commonly used in the filed of wastewater treatment and being under investigation for their antimicrobial properties is layered double hydroxides (LDHs)^19^.

LDHs are 2D anionic clay materials with a structure similar to brucite and chemical formula [M(II)(1 − x)M(III)x(OH)2]^x+^(A^n^−)_x/n_.yH_2_O. M(II) is a divalent cation such as Mg, Ni, Zn, Cu or Co and M(III) is a trivalent cation such as Al, Cr, Fe or Ga, while A^n^- is negative anions such as CO_3_^–^ , Cl^–^, NO_3_^–^ or organic anions^20,21^. LDH are very promising candidates for several applications such as (photo)-catalysis, catalysis, nano-adsorbents, and energy storage materials. In the field of catalysis, LDH samples have shown promising performance for several reactions including carbon dioixde conversion, water splitting, and poluutant degradation. As nano-adsorbents, LDH samples showed high removal effecicienec for numerous pollutants in wastewater effluents such as heacy metals, phenols, and dyes. For energy storage, LDH emerged as promsing candidates for batteris and supercapacitor applications. In additon, LDH samples were identified as possible antimicrobial agents against several pathogens. Several mechanisms were reported to explain the antibacterial perfomance of LDH samples. These mechanisms include adsorpting to the negatively charged bacteria cell wall, metal ions release from the LDH layers and generation of reactive oxygen species^22–24^. In addition, LDH showed mulitfunctionality in the field of wastewater treatment besides their antibacterial properties. Multi-functionality is an important criterion for nanomaterials applied in wastewater treatment. This is because multi-fucntional materials can be used to achieve several goals such as wastewater disinfection, pollutant adsorption, and organic molecules photo-degradation. Recently, Sharma et al. reviewed the reported studies investigating the multifunctional applications of LDHs in wastewater treatment^25^. The authors showed that LDHs can be used as photocatalysts, nano-adsorbents, and antibacterial agents. Therefore, LDH samples that showed high performance towards the adsorption of certain pollutants main gain even extra potential for real-life applications if proven as multifunctional samples. This opened the door towards the exploration and the investigation of LDH use in numerous wastewater related applications to assess their multifunctionality.

Our research group has investigated numerous LDH samples for their multifunctionality in the field of wastewater treatment. Moaty et al. prepared nitrate intercalated ZnFe LDH using a simple coprecipitation technique^26^. The prepared LDH showed high removal percent for heavy metals in wastewater along with excellent antimicrobial behavior. Amin et al. prepared Gamma irradiated CoFe LDH and reported good antibacterial activity against both Gram-positive and Gram-negative bacteria strains along with high removal of malachite green (MG) and methylene blue (MB) dyes^27^. Zaher et al. reported the promising antibacterial properties and oxytetracycline hydrochloride removal capacities of ZnFe LDH nano-adsorbent^28^. Sayed et al. prepared Co and Ni double-substituted ZnFe LDH and investigated its capacity for the removal of MO along with its antibacterial properties^29^. Moreover, LDH nanocomposites were also investigated to assess their multifunctionality. Mahmoud et al. TiFe LDH / chitosan nanocomposite using the milling technique^30^. The prepared composite showed high removal percentages against phosphate, cadmium, and benzoquinone in wastewater streams along with promising antibacterial properties.

On the other hand, more studies are required to investigate and optimize the performance of such LDH samples towards higher removal percentages of pollutants and better antibacterial performance. In this study, three types of ZnFe LDH samples were prepared using facile co-precipitation technique. These samples showed very promising performance as adsorbents for pollutants in simulated wastewater effleunts (manuscript under publication). This work aims at investigating the multifunctionality of these samples as nanomaterial based disenfectants to control microbial growth found in real dairy wastewater. No previous study has considered these LDH structures as multifunctional materials for wastewater disenfection applications. The first LDH sample is Co subsitituted ZnFe LDH with Zn equally subsitituted to Co. The sample is named CoZnFe LDH. Similarly, the second sample is Mg substituted ZnFe LDH named MgZnFe. Finally, MgZnFe was prepared along with triazol compound (3-amino-1H-1,2,4- triazole) which is named MgZnFe-Tz. Such composite between the MgZnFe and triazol showed promising biomedical applications as reported in our recent work (under publication). All samples were tested for their antibacterial properties for pathogens in dairy wastewater samples collected from a local farm (Fayoum, Egypt).

## Materials and methods

### Sample collection

A cross-sectional study was performed during the period from May to September 2021 in which seventy five wastewater samples were collected from a local dairy farm sewage system. Wastewater samples, which are alkaline in nature, were collected in sterile transparent glass containers, where the containers were sterilized in a hot air oven before collection. At the collection point, containers were rinsed several times with the water to be collected, filled, corked tightly and then labeled and sent to the laboratory of Animal Hygiene and Zoonoses, Faculty of Veterinary Medicine, Beni Suef University in ice box and when required were stored transiently at 4 °C. All samples were examined immediatley after being recieved to avoid any possible physico-chemical changes in the wastewater samples^31^.

### Isolation and identification of *Staphylococcus* and *Streptococcus*

Each wastewater sample was directly cultured on the surface of sodium azide crystal violet blood agar (Oxoid, CM0259) and Mannitol Salt agar (MSA) media (Oxoid, CM0085) for isolation of *Staphylococcus* and *Streptococcus* species, respectively. All plates incubated at 37˚C for 24–48 h. The colours and morphologies of the colonies were noted from the selective plates followed by the biochemical tests for identification of *Staphylococcus* and *Streptococcus* species^32^.

### Molecular identification of the isolates

Firstly, DNA was individually extracted from each sample using QIAamp DNA Mini kit (Qiagen, Germany, GmbH). Briefly, 200 µl of the sample suspension was incubated with both 10 µl of proteinase K and 200 µl of lysis buffer at 56 °C for 10 min. After incubation, then 200 µl of the absolute ethanol was added up to the lysate. The samples were washed and centrifuged following the manufacturer’s instructions. Lastly, the nucleic acid of each sample was eluted with 100 µl of elution buffer supplied with the kit. For PCR amplification, primers were utilized in a 25 µl volume reaction including 12.5 µl of Emerald Amp Max PCR Master Mix (Takara, Japan), 1 µl of each primer of 20 pmol concentration, 4.5 µl of water as well as 6 µl of DNA template. The reaction was performed in an applied biosystem 2720 thermal cycler. The PCR amplification of both S. *agalactiae*, S. *dysgalactiae* specific 16S rDNA, 23S rRNA identification gene specific for *Staph*. *aureus* as well as the gene responsible for QAC disinfectant resistance *(QacED1)* were listed in Table 1. The thermocycling parameter commenced as follows, an initial denaturation cycle at 94 °C for 5 min followed by 30 cycles of the consequent schedule, 94 °C for 30 s. The annealing temperatures were 60, 60, 55 and 58 °C for 45 s for each primer^33–36^, respectively. The final extension step was at 72 °C for 7 min. The products of PCR were separated by gel electrophoresis on 1.0% agarose gel (Applichem, Germany, GmbH) in 1 × Tris/Borate/EDTA (TBE) buffer at room temperature using gradients of 5 V/cm. For gel evaluation, 40 µl of the PCR products was inserted in each gel hole and the gel was interpreted by a gel documentation scheme (Alpha Innotech, Biometra).

**Table 1:** Primers sequences, target genes, amplicon sizes for the isolated bacterial species.

Target bacteria	Target gene	Primers sequences	Amplified segment (bp)	References
S. agalactiae	16S rRNA	CGCTGAGGTTTGGTGTTTACA	405	Mashouf et al., 2014
		CACTCCTACCAACGTTCTTC		
S. dysgalactiae		GGGAGTGGAAAATCCACCAT	572	Shome et al., 2012
		AAGGGAAAGCCTATCTCTAGACC		
S. aureus	23S rRNA	AC GGAGTTACAAAGGACGAC	1250	Bhati et al., 2016
		AGCTCAGCCTTAACGAGTAC		
S. aureus, S. agalactiae and S. dysgalactiae	QacED1	TAA GCC CTA CAC AAA TTG GGA GAT AT	362	Chuanchuen et al., 2007
		GCC TCC GCA GCG ACT TCC ACG		

### In-vitro evaluation of disinfectants and prepared nano-material efficacy against the isolated bacteria

The biocidal power of two commercially used disinfectants in water treatment were approved by the food industry^37^; Terminator (glutaraldehyde(150 g/L) + quat. ammonium chloride(100 g/L)), TH4 (is a combination of 4 quaternary ammonium, glutaraldehyde and 2 terperne derivatives), both disinfectants were obtained from 6th October 3rd Industrial Area, Egypt. The disinfectants were tested using different concentrations of them against 60 strains of s. agalactia, s. dysgalactia and staph. aureus isolated from the wastewater samples using broth agar well-diffsuion method^38^.

### Antibacterial activity assay

Antibacterial activity of all samples was assessed on all the identified bacterial isolates using agar well diffusion technique^39^. Inoculum containing 10^6^ CFU/ml of each bacterial culture to be tested was spread on the surface of nutrient agar plates with a sterile swab moistened with the bacterial suspension. Subsequently, wells of 6 mm diameter were cut into the agar medium using sterile micropipette tips and filled with 40–50 μl of the tested materials and allowed to diffuse at room temperature for 2 h. The plates were then incubated in the upright position at 37° for 24 h. Wells containing the same volume of DMSO (10.0%), and distilled water served as negative controls. After incubation, the diameters of the growth inhibition zones were measured and interpreted.

### Synthesis of CoZnFe LDH

Zinc nitrate (Chem-Lab NV -Belgium), Cobalt nitrate and Ferric nitrate (Oxford -India) were used as received without any further purification. Sodium hydroxide (NaOH) was purchased from Egyptian Piochem for laboratory chemicals. The co-precipitation method was used to prepare ZnCoFe (LDH) following a procedure similar to our previous work^39^. The ratio of Zn nitrate to Cobalt nitrate to Ferric nitrate used was adjusted to be 1.5:1.5:1 by mole respectively. Briefly, metal nitrates were precipitated using a slow addition (0.1 mL/min) of NaOH solution (2 M) until the solution pH reached 9 to assure complete precipitation of the corresponding metal hydroxides. To follow, the precipitated hydroxides were left under continuous stirring overnight to age. The formed suspension was filtered and washed using distilled water and finally washed with ethanol.

### Synthesis of MgZnFe LDH and MgZnFe-Tz LDH

Magnesium nitrate was purchased from Alpha Chemika (India). The ratio of Mg nitrate to Zn nitrate to Ferric nitrate used was adjusted to be 2:2:1 respectively. The co-precipitation technique was used to prepare MgZnFe LDH similar to that of CoZnFe LDH. To prepare MgZnFe-Tz LDH, the metal nitrates were precipitated in the presence of triazol (3-amino-1H-1,2,4- triazole) with concentration of 0.1 M in solution. The same procedure was followed as already mentioned for CoZnFe LDH.

### Material characterization

The prepared ZnCoFe MMO was characterized using different tools: PANalytical (Empyrean) X-ray diffractometer with Cu-Kα radiation (wavelength 0.154 nm, current = 35 mA, voltage = 40 kV, scanning at rate of 8° min^−1^) from two-theta of 5° to 80° was used to determine the crystallinity of the sample. The functional groups were determined using Fourier transform infrared (FTIR) spectroscopy (Bruker-Vertex 70, KBr pellet technique, Germany), from 400 to 4000 cm^−1^ wavenumber. The microstructure and morphology of the synthesized ZnCoFe MMO were investigated using Field Emission Scanning Electron Microscope (FESEM). CTX and Malachite green concentrations were measured using ultraviolet and visible light UV–VIS spectrophotometer (SHIMADZU UV-2600). An atomic absorption spectrophotometer (AAS) (model ZEISS-AA55, Germany) was used to detect the concentrations of elements in solutions.

### Ethical approval

This manuscript does not involve human participants, human data, or human tissue.

### Consent to participate

The manuscript does not contain any individual person’s data.

## Results and discussion

### Material characterization

**Figure 1:**
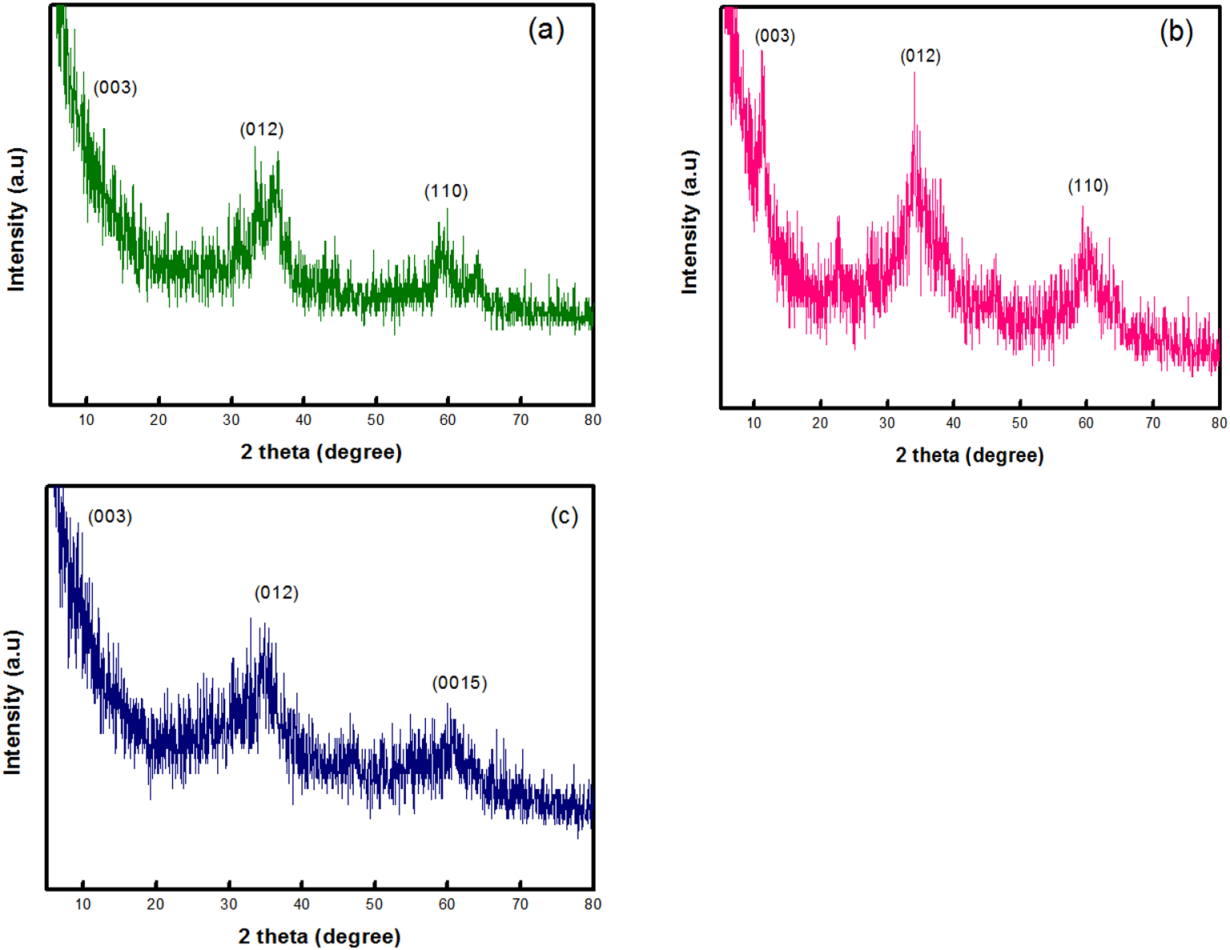
XRD diffractogram of (**a**) CoZnFe LDH, (**b**) MgZnFe LDH, and (**c**) MgZnFe-Tz LDH.

The XRD diffractograms of all prepared samples are shown in Fig. 1. Generally, Layered double hydroxide materials show diffraction peaks at 11.6°, 23.2°, 34.5°, 39.1°, 46.5°, 59.9°, and 60.9° that could be indexed to the plane families (003), (006), (012), (015), (018), (110), and (0015) respectively^40^. As shown in Fig. 1a, CoZnFe LDH shows a clear semi-crystalline nature probably due **to the nature of the preparation technique. Similarly, MgZnFe LDH and MgZnFe-Tz LDH (Fig. 1b,c respectively) shows similar behavior. The crystallite sizes of the CoZnFe LDH, MgZnFe LDH, and MgZnFe-Tz LDH samples were 7.48, 9.93 and 3.73 nm respectively. The decrease in the crystallite size after the addition of the triazol compound may be attributed to the capping of this compound to the layers of the MgZnFe LDH phase therefore preventing long-range order of such layers during the aging step of the precipitated LDH phase.

**Figure 2:**
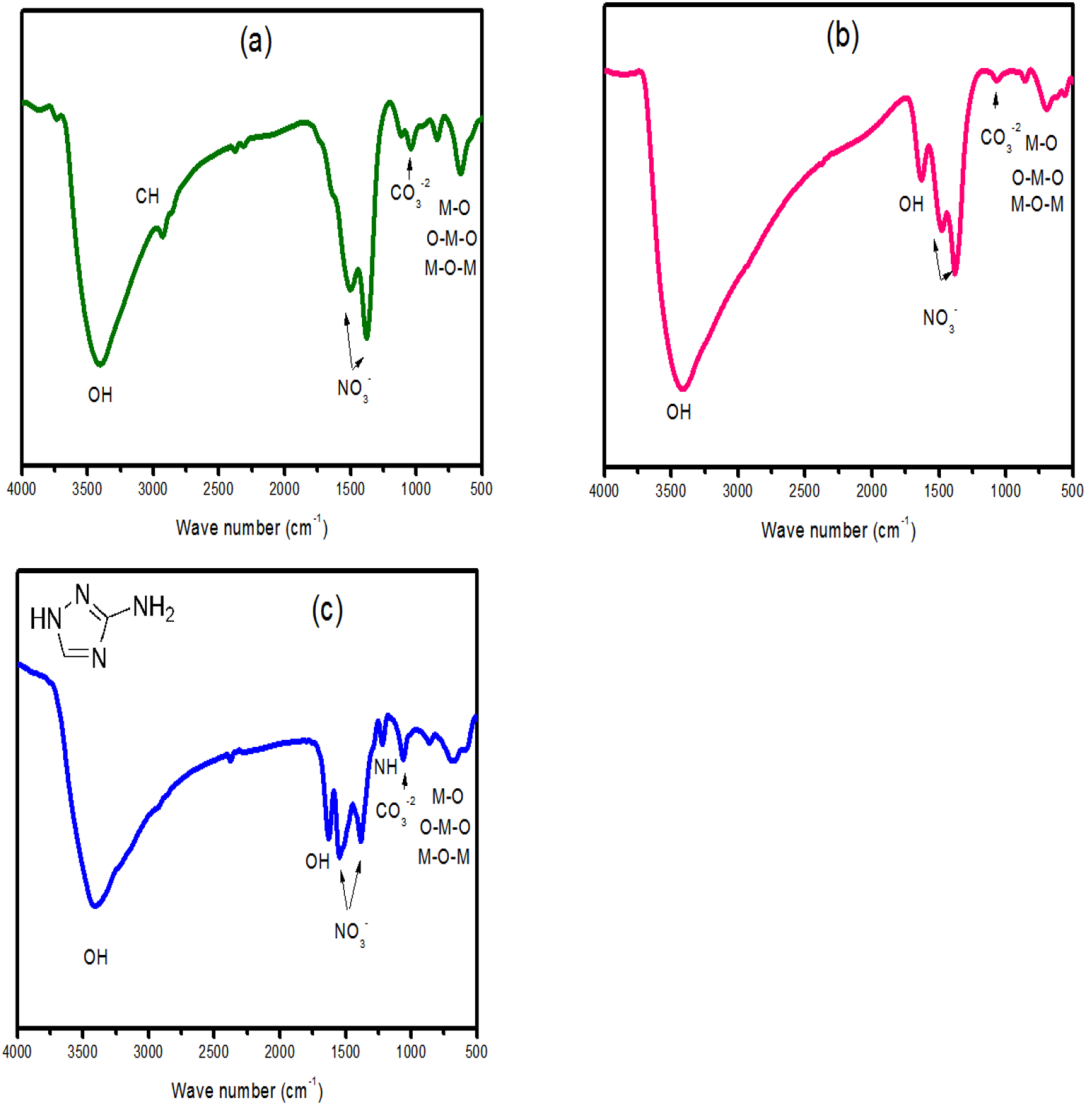
FTIR spectra of (**a**) CoZnFe LDH, (**b**) MgZnFe LDH, and (**c**) MgZnFe-Tz LDH.

FTIR spectra of all samples are shown in Fig. 2. As shown in Fig. 2a, the FTIR spectrum of the CoZnFe LDH sample shows a broad peak around 3400 cm^−1^, which can be ascribed to the OH^−^ stretching due to adsorbed water molecules^39^. Two sharp peaks at around 1510 and 1368 cm^−1^ can be attributed to the stretching mode of the nitrate ions^41^. The small peak at around 2900 cm^−1^ may originate from ethanol molecules used during the washing step^42^. Below 1000 cm^−1^ the observed peaks resulted from the metal oxide (O–M–O, M–O, M–O-M) vibrations in the clay-like layers^43,44^. These bonds originate from the di or tri metal center in the octahedron structures forming the LDH layers. The MgZnFe sample shows similar FTIR spectra to CoZnFe sample with one extra peak at 1630 cm^−1^ is can be attributed to the bending vibration of the interlayer H_2_O water molecules^45,46^. FTIR spectra of MgZnFe-Tz was similar to that of MgZnFe LDH with extra peak at 1230 cm^−1^ originating from the N–H bending of the amino group in the triazol (3-amino-1H-1,2,4- triazole) compound^47^.

**Figure 3:**
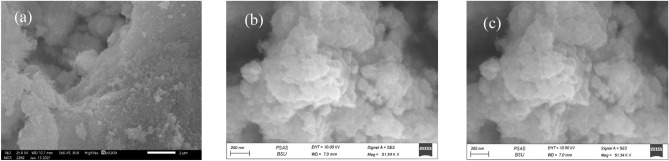
SEM images of (**a**) CoZnFe LDH, (**b**) MgZnFe LDH, and (**c**) MgZnFe-Tz LDH.

SEM images of all samples are shown in Fig. 3. CoZnFe LDH shows a typical layered structure as presented in Fig. 3a. Similarly MgZnFe LDH showed a similar morphology as shown in Fig. 3b. No change in morphology for the MgZnFe-Tz sample (Fig. 3c) was observed as compared to the MgZnFe LDH sample. Figure S1 shows TEM images for the prepared samples. These images show that the prepared LDH has a wide layer size distribution. This is attributed to the co-precipitation method that lacks control over the layer size while being a cost effective and simple preparation method suitable for practical applications.

**Figure 4:**
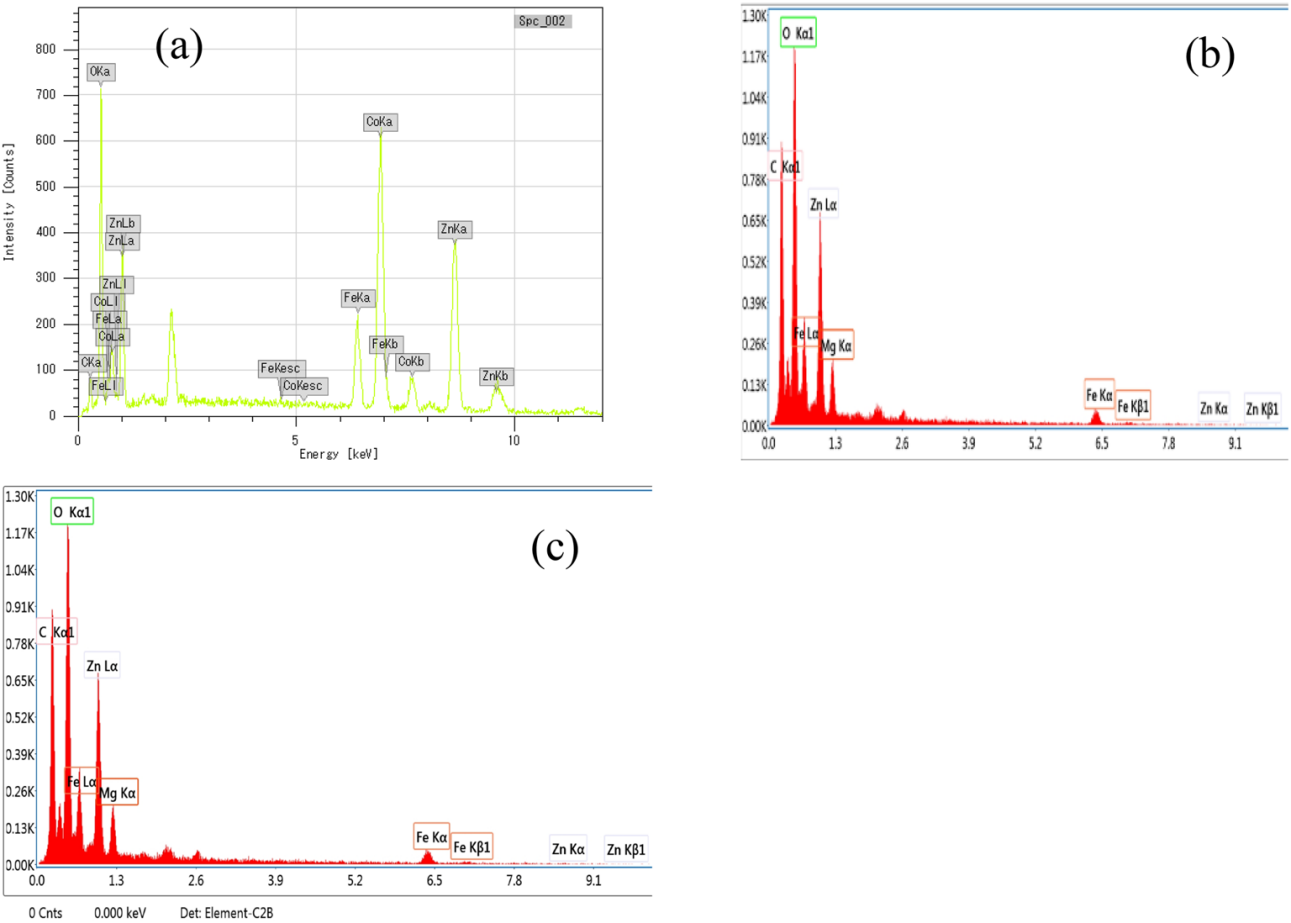
Edx analysis of (**a**) CoZnFe LDH, (**b**) MgZnFe LDH, and (**c**) MgZnFe-Tz LDH.

Edx analysis of the samples is shown in Fig. 4. All Edx spectra did not show any foreign species, thereby reflecting the purity of the prepared samples. Sample CoZnFe LDH (Fig. 4a) showed peaks for Co, Zn and Fe. Similarly, MgZnFe LDH and MgZnFe-Tz (Fig. 4b,c respectively) showed peaks for Mg, Zn and Fe only.

**Figure 5:**
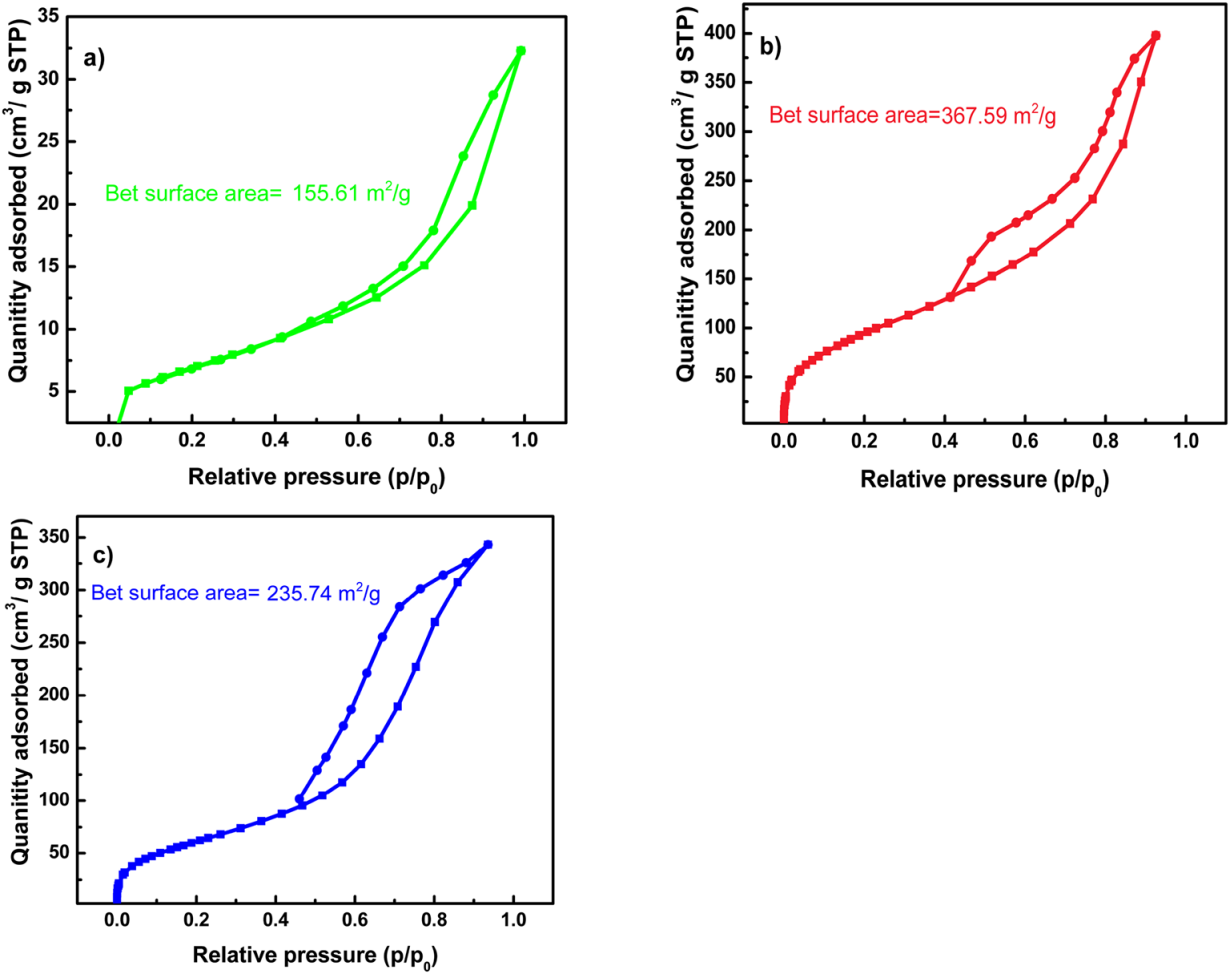
Nitrogen adsorption desorption isotherms of (**a**) CoZnFe LDH, (**b**) MgZnFe LDH, and (**c**) MgZnFe-Tz LDH.

Figure 5 represents the nitrogen adsorption desorption isotherms of all samples. All isotherms can be classified as type IV isotherm with hysteresis loop of types H3^48^. Type IV isotherm originates from samples with mesoporous structures. Mesoporous materials are those with pore widths ranging between 2 and 50 nm^48^. Moreover, H3 hysteresis loops originate from non-rigid aggregates of plate-like particles^48^, which is the case for all the LDH samples. LDH samples has layered structure where the divalent and trivalent cations from alternative octahedrons where the oxygen atoms are bonded at the corners and the metal cation in the center. These octahedrons form the layered structure of the LDH samples leading to the H3 type loop in the nitrogen adsorption–desorption isotherm. Table 2 shows the BET surface area, pore volume and mean pore diameter of all samples. As shown MgZnFe LDH has almost 2.3 folds the area of CoZnFe LDH probably because of the larger crystallite size. After Tz addition, the surface area decreased by about 35% owing to the adsorption of Tz on the LDH surface.

**Table 2:** BET surface area, pore volume and mean pore diameter of all samples.

Sample	CoZnFe LDH	MgZnFe LDH	MgZnFe-Tz LDH
BET surface area (m^2^/g)	155.61	367.59	235.74
Total pore volume (cm^3^/g)	0.26	0.62	0.53
Mean pore diameter (nm)	3.82	6.69	9

### Antimicrobial study

**Figure 6:**
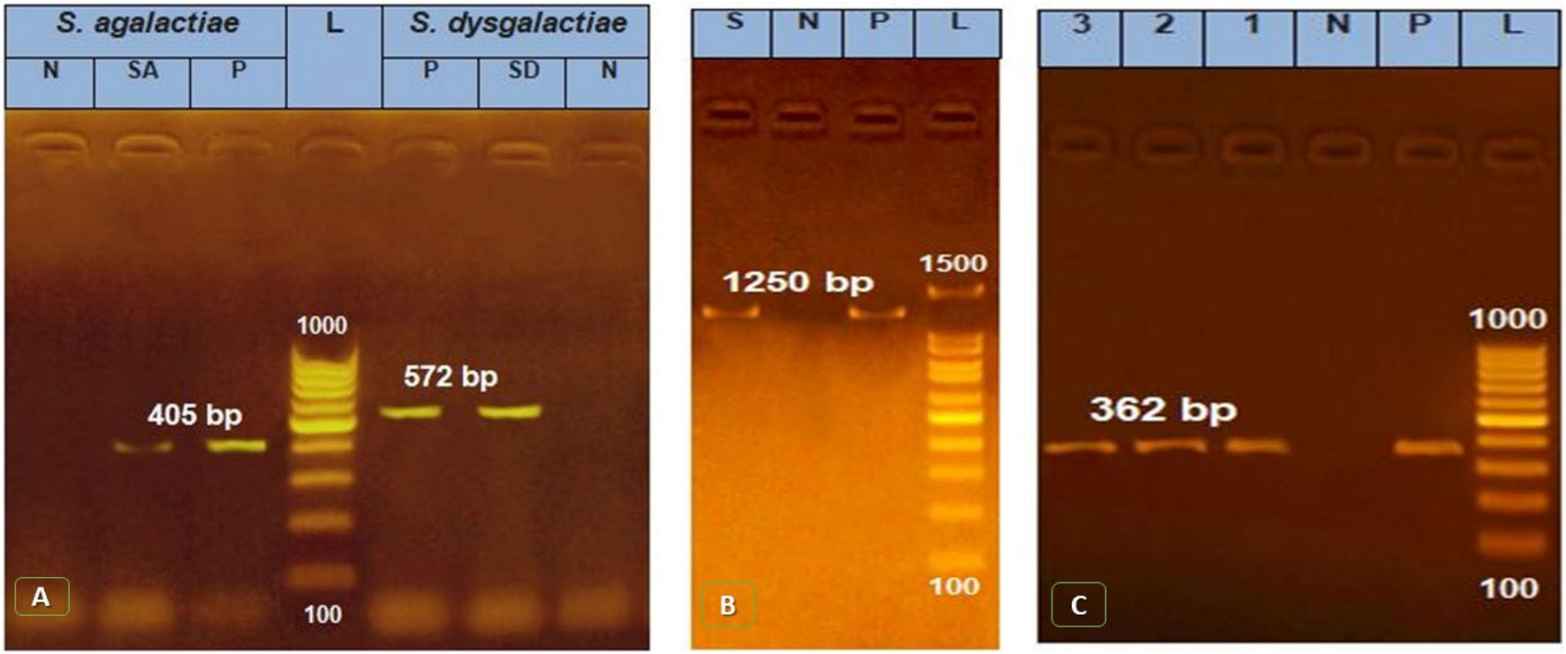
Agarose gel electrophoresis for PCR products of **16S rRNA** specific for S. agalactia and S. dysgalactia (**A**) amplified 405 bp and 572 bp, respectively, also **23S rRNA** gene specific for Staph. aureus (**B**) amplified 1250 bp and the QAC disinfectant resistance gene (QacED1) amplified 362 bp (**C**). Lane (L): 100 bp Ladder ‘’Marker’’, Lane: (1–10), the examined samples, Lane Pos: Positive control, Lane Neg: Negative control.

The presence of microbial pathogens in dairy farms' wastewater might cause the contamination of raw milk with these pathogens through wastewater one way or another, which might be of major public health significance therefore, it became a need to control these pathogens. Results in Table 3 the prevalence of some pathogens of zoonotic importance that might be found in wastewater such *Staph. aureus S. agalactica* and *S. dysaglactica* it showed high prevalence (80.0%) of collected samples were positive for microbial isolation. *Staph. aureus* was the dominant pathogen to be isolated (41.3%) followed by *S. dysaglactica* and *S. agalactica* (22.7 and 16.0%, respectively) this was statistically significant (P > 0.05). the isolated strains of *Staph. aureus S. agalactica* and *S. dysaglactica* were genetically assayed to detect *16S rRNA* gene specific for *S. agalactia* and *S. dysgalactia* amplified 405 bp and 572 bp, respectively (Fig. 6 A), also *23S rRNA* gene specific for *Staph. aureus* (B) amplified 1250 bp (Fig. 6B).

**Table 3:** Distribution of the isolated bacteria from the examined wastewater samples.

Wastewater sample	S. agalactiae		S. dysgalactiae		Staph. aureus		Total	%
	No. positive	%	No. positive	%	No. positive	%		
N = 75	12	16.0	17	22.7	31	41.3	60	80.0
P- value = 0.008, X2 = 9.700

Moges et al.^49^ reported similar results to the findings in our study where he mentioned that (85.0%) of the collected wastewater samples were positive for one or two pathogens but recorded much lower isolation rate to *S. aureus* (8.2%) than our findings as well, Ben Said et al.^50^ isolated *S. aureus* by (19.35%). Also, Jankovic et al. (2020) mentioned that the isolation of *Staphylococcus* was by 21.0%. Amal et al.^51^ performed a study on fish cultured in lack ponds located near residential areas and agricultural waste disposal sites (water of low quality). The authors found the isolation of *S. agalactica* was (10.42%) which is nearly similar to our findings. In this study, the teamwork was concentrating on pathogens which are mainly related to dairy animals and industry, such *S. dysaglactica, S. agalactica* and *Staph. aureus* and we contribute the presence of these pathogens in the farm wastewater due to the measures being taken in the farm under the study like throwing contaminated milk and animal wastes in the farm sewer system.

Results illustrated in Table 4 showed that the sensitivity of the isolated strains of *S. dysaglactica, S. agalactica* and *Staph. aureus* was significantly high to the commonly used disinfectants in water treatment at different concentrations. Whereas there were variable degrees of resistance to the nanoparticles used, as it was cleared that CoZnFe LDH showed a significant efficacy in combating of *S. dysaglactica, S. agalactica* and *Staph. aureus* isolates followed by MgZnFe LDH and the lowest biocidal potency was showed by MgZnFe-Tz LDH. According to our findings, it was revealed that the elevated resistance rate to the used disinfectants was confirmed by determination of the *QacED1* gene (Fig. 6C) responsible for the resistance to quaternary ammonia compound, the main constitute in the used disinfectants.

**Table 4:** Comparison of the biocidal effect of the used disinfectants and nanoparticles against the isolated bacteria from the examined wastewater samples.

Chemicals used	Concentration (mg/L)	S. Agalactiae			S. dysgalactiae			Staph. aureus		
		S	I	R	S	I	R	S	I	R
Terminator	0.3	7.0	8.0	85.0	3.0	9.0	88.0	4.0	9.0	87.0
	0.5	11.0	12.0	77.0	9.0	15.0	76.0	13.0	15.0	72.0
TH4	1:200	7.0	2.0	92.0	0.0	12.0	88.0	17.0	19.0	64.0
	1:400	14.0	6.0	80.0	19.0	10.0	81.0	23.0	19.0	58.0
Co–Zn–Fe nanoparticles	0.01	45.0	19.0	36.0	39.0	22.0	39.0	33.0	29.0	52.0
	0.05	62.0	22.0	16.0	49.0	33.0	18.0	51.0	34.0	15.0
Zn- Fe-Co–Ni nanoparticles	0.01	32.0	10.0	58.0	30.0	7.0	63.0	22.0	9.0	69.0
	0.05	40.0	12.0	48.0	37.0	10.0	53.0	29.0	11.0	60.0
MN-Fe-Zn-Trizol nanoparticles	0.01	20.0	0.0	80.0	25.0	5.0	70.0	19.0	4.0	77.0
	0.05	25.0	5.0	70.0	22.0	7.0	71.0	30.0	4.0	66.0
P-value		0.000			0.000			0.001		

Based on our findings, it was evident that LDH had a strong antibacterial effect on the screened microbes because bacteria were able to stick to and adsorb to its surface via electrostatic forces produced by the metallic ion exchanged LDH, leading to higher antibacterial efficacies^52,53^. Recent years have seen a lot of interest in LDH's antibacterial capabilities. LDH toxicity was evaluated by^54^ against *Streptococcus*, with an EC50 of 10 mg/L following 72 h of LDH exposure. Additionally, the LDH concentration of 50 mg/L fully prevented the development of *Streptococcus*. Also, LDH exhibited a long-lasting antibacterial activity against both Gram-positive bacteria (*Staph. epidermidis, S. pyogenes, and Staph aureus*) as well as Gram-negative bacteria (*Proteus vulgaris, Klebsiella pneumoniae, Escherichia coli, Pseudomonas aeruginosa,* and *Salmonella*) as reported previously^26,55^.

**Figure 7:**
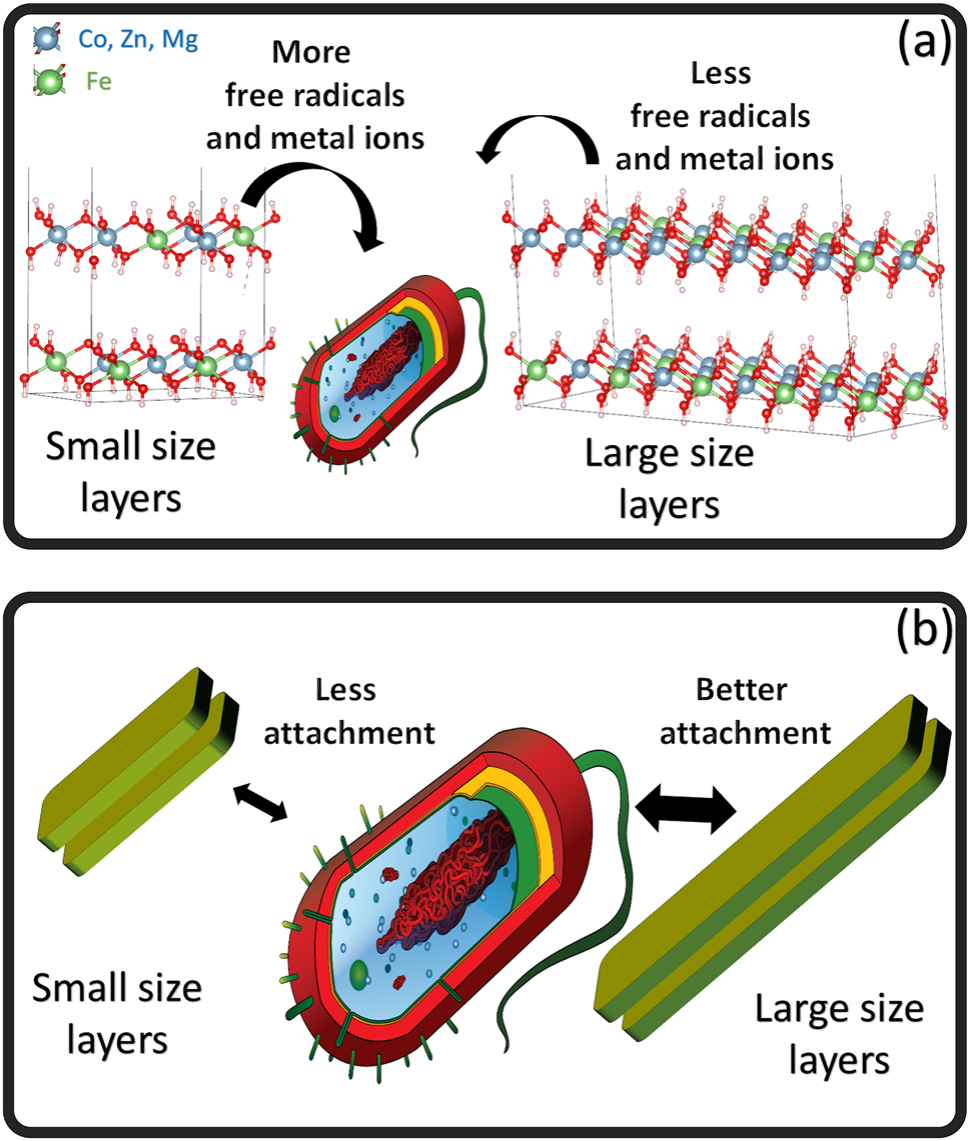
(**a**) free-radical mechanism and (**b**) attachment mechanism of LDH on bacteria cells showing the effect of small and large sized layers in the samples. (bacteria cell image was downloaded from pixabay.com).

It was obvious that all the examined microorganisms displayed an *In-vitro* considerable antibacterial activity against Zn–Fe LDH. This could be explained by the release of hydroxyl ions from the Zn–Fe LDH in a wastewater setting. These hydroxyl ions are extremely reactive free radicals that have the potential to damage a variety of biomolecules, including the DNA and cytoplasmic membrane of bacteria as well as cause protein denaturation. These free radicals are believed to be released easier from layers of small layer sizes (Fig. 7a). These layers are believed to be easily dissolve compared to layers of large layer size. Additionally, the existence of various metal mixtures in LDH like Fe, Ni, Zn, Co are usefully reactive with proteins and preventing the controlled transfer through the plasma membrane by permeability affecting the transport system and lead to the death of bacteria^56,57^.

On the other hand, layer size can affect the attachment on the bacteria cell membrane. Zn–Fe LDH's have positive charge with zeta potential values up to 30–50 mV, improving its antibacterial action^58^. This may be explained by the abundant holes and high peptide poly-glycogen content of Gram-positive bacteria's cell wall, which made it easy for foreign molecules to enter the cell and facilitated the deadly effect-producing faster ion absorption^59^. It is believed that layers with larger sizes can contribute positively to the attachment mechanism compared to smaller sizes (Fig. 7b). To sum up, the prepared samples have a wide layer size distribution. Small layers easily dissolve and produce free radicals and metal ions. On the other hand, large layers easily attach on cell membrane causing membrane disruption and deterioration. Both size contribute to the observable antibacterial effect of the prepared LDH samples.

## Conclusion

Since there is a global trend toward using treated wastewater and due to the scarcity of water resources all over the word, therefore it became a need to search for alternatives to traditionally used materials that are used in the treatment of different contaminants in wastewater such bacteria, heavy metals and antibiotics. Three Zn–Fe based LDH samples were successfully prepared and characterized using XRD, FTIR, SEM, BET and Edx. The prepared samples were tested for their antibacterial properties against pathogens in real dairy wastewater samples. From the current study, it was concluded that *S. agalactia*e, *S. dysgalactiae* and *Staph. aureus* prevailed in dairy farm wastewater sources. The lack of proper sanitation for such wastewater effluents will result in the in contamination of dairy products. The use untreated wastewater can result in the infection of dairy animals and/or their products through the capacity of the pathogens that could gain access to human food chain through the consumption of contaminated products. Due to the acquired resistance of most pathogens to commonly used disinfectants. Therefore, it is important to regularly clean and remove organic matter from entering the animal’s water sources and passing through the entire system of dairy production. The better efficacy of CoZnFe LDH has been demonstrated in this study, making them a promising material in controlling the zoonotic pathogens in water sources to improve the dairy industry.

## Supplementary Information

Supplementary Information.

## Data Availability

The datasets used and/or analysed during the current study available from the corresponding author on reasonable request.
