# Supplementary material for: Antimicrobial properties of promising Zn–Fe based layered double hydroxides for the disinfection of real dairy wastewater effluents
Source: Sci Rep. 2023 May 10;13:7601. doi: 10.1038/s41598-023-34488-y (PMC10172331; doi:10.1038/s41598-023-34488-y)
Supplement: Supplementary file 1 — Supplementary Information. [file 41598_2023_34488_MOESM1_ESM.docx]

**Supporting information**

Antimicrobial properties of promising Zn-Fe based layered double hydroxides for the disinfection of real diary wastewater effluents

Sahar Abdel Aleem Abdel Aziz^1^, Yasser GadelHak ^2^, Manar Bahaa El Din Mohamed^1^, Rehab Mahmoud^3*^

1 Department of Hygiene, Zoonoses and Epidemiology, Faculty of Veterinary Medicine, Beni–Suef University, 62511Beni–Suef, Egypt. [sahr.ismail@vet.bsu.edu.eg](mailto:sahr.ismail@vet.bsu.edu.eg), [dr.manarbahaa@gmail.com](mailto:dr.manarbahaa@gmail.com)

2 Department of Materials Science and Nanotechnology, Faculty of Postgraduate Studies for Advanced Sciences, Beni-Suef University, Beni-Suef 62511, Egypt yasser191919@gmail.com

3 Department of Chemistry, Faculty of Science, Beni-Suef University, 62511 Beni-Suef, Egypt. [prof.rehab.mahmoud@gmail.com](mailto:prof.rehab.mahmoud@gmail.com)


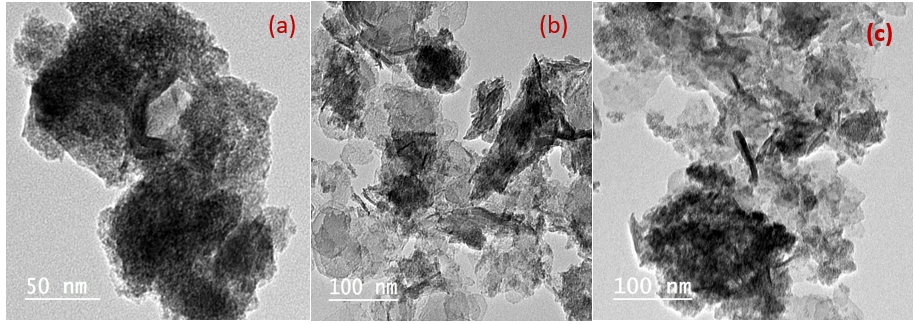


Figure S1: TEM of (a) CoZnFe LDH, (b) MgZnFe LDH, and (c) MgZnFe-Tz LDH.


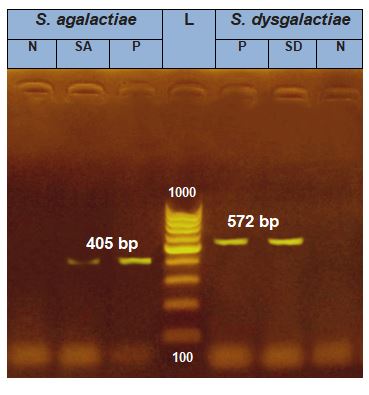


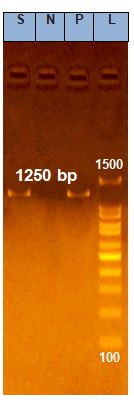


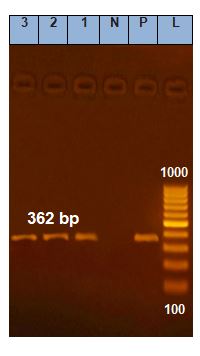


Full Figure 6 Agarose gel electrophoresis for PCR products of **16S rRNA** specific for S. agalactia and S. dysgalactia (A) amplified 405 bp and 572 bp, respectively, also **23S rRNA** gene specific for Staph. aureus (B) amplified 1250 bp and the QAC disinfectant resistance gene (QacED1) amplified 362 bp (C). Lane (L): 100 bp Ladder ‘’Marker’’, Lane): 1-10), the examined samples, Lane Pos: Positive control, Lane Neg: Negative control.
